# 

**Published:** 1908-02-08

**Authors:** 


### BOOKS RECEIVED.

Baillière, Tindall, and Cox.

"Aids to Pathology." By H. Campbell, M.D.

"The Hair and its Diseases." 2nd edition. By David Walsh, M.D.

"Medical Laboratory, Methods, and Tests." 2nd edition. By Herbert French, M.D.

"Aids to Anatomy." 2nd edition. By Joseph Cunning[illegible], F.R.C.S.

"The Pocket Anatomy." 6th edition. Edited by C. H. Fagge, F.R.C.S.

The Scientific Press, Ltd.

"The Matron, Her Duties and Responsibilities."

Wells Gardner, Darton and Co., Ltd.

"The Book of Simple Remedies." By Florence Stacpoole.

J. Wright and Co., Ltd., Bristol.

"Index of Treatment." By R. Hutchison, M.D., and H. Stansfield Collier, F.R.C.S.

A. Constable and Co., Ltd.

"Essentials of Cytology." By C. E. Walker.

### THE GRADUATES' MEDICAL SCHOOLS.

### Diary for the week February 10 to 15.

**MEDICAL GRADUATES' COLLEGE AND POLYCLINIC, Chenies Street, W.C.**

**Lectures at 5.15 p.m.**

**Feb. 10,** Dr. Purves Stewart, **Thalamic Hemiplegia.**
**Feb. 11,** Dr. M. Dobbie, **Swedish Medical Gymnastics; their Application in the Treatment of Diseases of the Circulatory and Respiratory Systems.**
**Feb. 12,** Mr. R. H. J. Swan, **Carcinoma of the Breast, its Early Recognition and Treatment.**
**Feb. 13,** Dr. J. E. Squire, **Pneumothorax.**

**LONDON SCHOOL OF CLINICAL MEDICINE, Seamen's Hospital, Greenwich.**

**At 3.30 p.m.**

**Feb. 12,** Mr. Cargill, **The Diagnosis of Errors of Refraction.**

**At 2.15 p.m.**

**Feb. 13,** Dr. Rankin, **Neurasthenia: Its Ætiology and Treatment.**

**NATIONAL HOSPITAL FOR THE PARALYSED AND EPILEPTIC, Queen Square, Bloomsbury, W.C.**

**Feb. 11,** Sir Wm. Gowers, F.R.S., **Clinical Lecture.**

**Feb. 14,** Dr. Batten, **Hereditary Ataxy.**

**CHARING CROSS HOSPITAL, W.C.**

**Feb. 13,** Mr. Boyd, **Surgical.**

**THE POST-GRADUATE COLLEGE, West London Hospital, Hammersmith, W.**

**At noon.**

**Feb. 10,** Dr. Low, **Pathological Demonstration.**

**Feb. 11 and 13,** Dr. Pritchard, **Practical Medicine.**

**At 5 p.m.**

**Feb. 10,** Dr. Davis, **Clinical, I.**

**Feb. 11,** Dr. Moullin, **Gynæcological.**

**Feb. 12,** Mr. Pardoe, **Cystitis.**

**Feb. 13,** Mr. Baldwin, **Practical Surgery, III.**

**At 3 p.m., at the London County Asylum, Claybury, Woodford Bridge, Essex.**

Dr. Robert Jones, **Types of Insanity.**

**THE THROAT HOSPITAL, Golden Square, W.**

**Feb. 10,** Dr. H. W. F. Powell, **Inflammation of the Mastoid Process—Acute or Chronic.**

**Feb. 13,** Dr. H. W. F. Powell, **Inflammation of the Mastoid Process—Abortive and Operative Treatment.**

**MOUNT VERNON HOSPITAL POST-GRADUATE COURSE, Hampstead.**

**Feb. 16,** Dr. T. N. Kelynack, **Demonstration of Methods of Treatment of Chest Cases.**

**THE HOSPITAL FOR SICK CHILDREN, Great Ormond Street, W.C.**

**At 4 p.m.**
**Feb. 13,** Mr. Corner, **Injuries and Diseases of the Epiphyses.**

### ANSWERS TO CORRESPONDENTS.

"Grateful."—The *Albion Magazine* is published by the Celtic Press, 38 Chancery Lane, London, W.C.

Dr. Renny.—An article on the method you mention will shortly appear.

H. B. Selby.—(1) "Furunculosis" will be dealt with in our columns. (2) The Insurance Manager is replying to this question.

THE HOSPITAL *February* 8, 1908.

*Name* ..............................................................................

*Address* ..............................................................................

..............................................................................................

This Coupon must accompany manuscript or contributions intended for The Hospital.

